# Combined Effects of Cyclic Hypoxic and Mechanical Stimuli on Human Bone Marrow Mesenchymal Stem Cell Differentiation: A New Approach to the Treatment of Bone Loss

**DOI:** 10.3390/jcm13195805

**Published:** 2024-09-28

**Authors:** Marta Camacho-Cardenosa, Victoria Pulido-Escribano, Bárbara Torrecillas-Baena, Jose Manuel Quesada-Gómez, Aura D. Herrera-Martínez, Rafael R. Sola-Guirado, Gabriel Dorado, María Ángeles Gálvez-Moreno, Antonio Casado-Díaz

**Affiliations:** 1 Unidad de Gestión Clínica de Endocrinología y Nutrición, Instituto Maimónides de Investigación Biomédica de Córdoba (IMIBIC), Hospital Universitario Reina Sofía, 14004 Córdoba, Spain; victoriapulido7@gmail.com (V.P.-E.); b42tobab@uco.es (B.T.-B.); jmquesada@uco.es (J.M.Q.-G.); aurita.dhm@gmail.com (A.D.H.-M.); mariaa.galvez.sspa@juntadeandalucia.es (M.Á.G.-M.); 2 Department Mecánica, Escuela Politécnica Superior, Universidad de Córdoba, 14071 Córdoba, Spain; ir2sogur@uco.es; 3 Department Bioquímica y Biología Molecular, Campus Rabanales C6-1-E17, Campus de Excelencia Internacional Agroalimentario (ceiA3), Universidad de Córdoba, 14071 Córdoba, Spain; bb1dopeg@uco.es; 4 CIBER Fragilidad y Envejecimiento Saludable (CIBERFES), Instituto de Salud Carlos III, 08003 Madrid, Spain

**Keywords:** hypoxia, mechanical stimuli, osteoblast, mesenchymal stem cells, adipocyte, bone

## Abstract

**Background:** The prevention and treatment of bone loss and osteoporotic fractures is a public health challenge. Combined with normobaric hypoxia, whole-body vibration has a high clinic potential in bone health and body composition. The effect of this therapy may be mediated by its action on bone marrow mesenchymal stem cells (MSCs). **Objectives:** Evaluate the effects of cyclic low-vibration stimuli and/or hypoxia on bone marrow-derived human MSC differentiation. **Methods:** MSCs were exposed four days per week, two hours/day, to hypoxia (3% O_2_) and/or vibration before they were induced to differentiate or during differentiation into osteoblasts or adipocytes. Gene and protein expression of osteoblastic, adipogenic, and cytoskeletal markers were studied, as well as extracellular matrix mineralization and lipid accumulation. **Results:** early osteoblastic markers increased in undifferentiated MSCs, pretreated in hypoxia and vibration. This pretreatment also increased mRNA levels of osteoblastic genes and beta-catenin protein in the early stages of differentiation into osteoblasts without increasing mineralization. When MSCs were exposed to vibration under hypoxia or normoxia during osteoblastic differentiation, mineralization increased with respect to cultures without vibrational stimuli. In MSCs differentiated into adipocytes, both in those pretreated as well as exposed to different conditions during differentiation, lipid formation decreased. Changes in adipogenic gene expression and increased beta-catenin protein were observed in cultures treated during differentiation. **Conclusions:** Exposure to cyclic hypoxia in combination with low-intensity vibratory stimuli had positive effects on osteoblastic differentiation and negative ones on adipogenesis of bone marrow-derived MSCs. These results suggest that in elderly or frail people with difficulty performing physical activity, exposure to normobaric cyclic hypoxia and low-density vibratory stimuli could improve bone metabolism and health.

## 1. Introduction

The prevention and treatment of osteoporosis and other bone conditions is a global public health challenge, which implies the need for more research to find non-pharmacological ways to reduce the current limitations of currently used drugs [1]. The most commonly used treatments include antiresorptive drugs and anabolic treatments, or sequential therapy with both [2]. These treatments have low adherence [3,4] and have rare but serious adverse effects, including atypical femoral fractures and osteonecrosis of the jaw [2].

Accumulating evidence indicates a complex relationship between bone marrow adiposity and osteoporosis [5]. Osteoblasts and adipocytes originate from the common-precursor mesenchymal-stem cells (MSCs), being the balance between differentiation of osteoblasts and adipocytes regulated by both intra- and extra-cellular factors [6]. Between others, aging alters the fate of MSCs in bone marrow by promoting adipogenesis and reducing osteoblastogenesis [7], and therefore increasing age-related bone loss [8].

Strength training has been widely recognized for its beneficial effects on different body systems [9]. However, these must be adapted to the characteristics of each patient, and, in elderly people, traditional methods may not be feasible. In this regard, whole body vibration (WBV) has been proposed as a viable, safe, and beneficial tool for this population [10]. On the other hand, it has been observed that hypoxia can affect bone metabolism. Decreased oxygen levels induce transcriptional activity of hypoxia-inducible factor 1 (HIF1). That regulates hundreds of hypoxia-adaptive genes, including the ones related to glucose metabolism, angiogenesis, cell differentiation, and apoptosis, among others [11]. At bone level, most studies indicate that sustained hypoxia over time decreases osteoblastogenesis and activates osteoclastogenesis, promoting bone loss.

Yet, interestingly, cyclic hypoxic exposure, in which short periods of exposure to moderate hypoxia (greater than 2% O_2_ in vitro and approximately 9 to 16% O_2_ in vivo) alternating with periods of normoxia, repeatedly for days or weeks, can improve bone formation, decreasing bone resorption [12]. In this sense, the combination of vibration-induced mechanical stimuli and hypoxic conditions could also enhance the physiological experience, exhibiting synergistic effects on age-related factors [13]. Similar physiological responses with less mechanical stress could be observed in hypoxic training compared to physical training alone, making it an interesting alternative for some populations [14]. Thus, therapeutic benefits of hypoxic training have been suggested for clinical populations such as older people [15]. Combined with normobaric hypoxia exposures, WBV appears to have effects, mediated by intervention characteristics, on bone health [16,17] and body composition [17]. Current research highlights the need to further ascertain the observed beneficial effects as well as the mechanisms responsible for these adaptations.

Hypoxia conditioning could modulate MSC proliferation and lifespan, with cyclic hypoxia (CH) holding potential for stem cell therapies [18,19,20]. Indeed, a recent review highlighted that MSCs preconditioning under hypoxic conditions may lead to upregulation of multipotency. Thus, expansion of MSCs under hypoxia followed by differentiation under normoxia can increase the differentiation potential compared to differentiation under normoxia or hypoxia alone. However, the duration of exposure to hypoxia and the O_2_ concentration required are yet to be analyzed [20]. Additionally, studies on the differentiation of MSCs into adipogenic and osteogenic lineages under hypoxic conditions have shown contradictory results. For instance, significant alterations of osteogenic and adipogenic differentiation under hypoxic conditions have been described by downregulation of osteogenic and adipogenic markers, respectively [21,22,23,24].

In contrast, other studies have observed increased osteogenic and adipogenic differentiation of MSCs under hypoxic conditions [25,26,27,28]. It should be taken into consideration that HIF1 controls angiogenic and osteogenic factors [29], bone remodeling-related genes, and glycolytic enzymes required for osteoblast differentiation [30]. That makes hypoxia a central regulator of bone formation. Indeed, mild/moderate cyclic hypoxia can trigger adaptive bone remodeling and differentiation of MSCs into osteoblasts [31,32]. Previous studies have established how the osteoprotegerin/receptor activator of nuclear factor kappa-Β ligand (RANKL) (OPG/RANKL) ratio increased in cultures of human MSCs, induced to differentiate in cyclic hypoxia (3% O_2_ for 1 or 2 h, four days per week). That was mainly due to a decrease of *RANKL* expression during periods of hypoxia [31,33,34]. In preclinical and clinical studies, CH conditioning inhibited bone resorption biomarkers, stimulating bone formation [17,35,36].

On the other hand, CH (3% O_2_ for 4 h cycles) decreased lipid droplet formation in MSCs induced to differentiate into adipocytes, repressing the expression of adipogenesis-related genes [31]. Murine C3H/10T1/2 MSCs lost capacity to differentiate into adipocytes after being preconditioned for 24 h with 0.1 mmol/L CoCl_2_, mimicking low-oxygen conditions [37]. These results showed changes in gene and metabolic expression in MSCs induced to differentiate into adipocytes, including downregulation of the peroxisome proliferator-activated receptor gamma coactivator 1-alpha (*PPARGC1A*) gene [31].

Interestingly, glycolytic metabolism predominates during hypoxia, inhibiting adipocyte maturation, as it negatively interferes with shifting towards oxidative metabolism. In addition, after CH conditioning, there is a continuous upregulation of Dickkopf Wnt signaling pathway inhibitor 1 (DKK1; Wnt is a portmanteau of Wg and int, standing for “wingless-related integration site”), which is an inhibitor of the beta-catenin pathway. That could prevent the correct maturation of adipocytes since their activation is required at the end of the adipocyte differentiation process [38]. Variation in experimental designs between studies, including time of exposure to hypoxia, O_2_ concentration, substrates, MSC tissue-culture factors, and other methodologies, could explain the contradictory reports.

On the other hand, MSCs in bone exist in a mechanically rich environment [39], to which they respond by means of numerous membrane proteins, cytoskeletal components, and nuclei. All of them may act as putative mechanosensors [40]. Interestingly, bones experience a small number of high-magnitude (~2000 to 3000 microstrains) low-frequency events (1–2 Hz) daily. They are bombarded by hundreds of smaller magnitude signals (<10 microstrains), being the result of high-frequency muscle contractions (10–50 Hz) [41]. During habitual loading, MSCs provide the necessary osteoblast populations to facilitate bone modeling, when bones are loaded beyond the physiological “sweet-spot” (~2000 to 3000 microstrains). That results in a net increase of bone formation [42].

Therefore, the rationale that exogenous application of low-magnitude mechanical signals, by using low-intensity vibration platforms, has been recognized as mechanical stimuli that can be anabolic and/or anti-catabolic to bone cells, both in vivo and in vitro [39,43,44]. Indeed, mechanical stimuli may promote osteogenic differentiation of bone-marrow MSCs [42,45], even in aged animal models [46], while pathways conducive to peroxisome proliferator-activated receptor gamma 2 (PPARG2)-driven adipogenesis are downregulated [42,47]. In contrast, inactivity or sedentarism increased marrow adipogenesis [48], increasing the expression of *PPARG2* and *RANKL* in MSCs, which also promotes osteoclast-mediated bone resorption. However, both are quickly suppressed by the introduction of mechanical stimuli [49]. A multitude of evidence suggests that the incorporation of multiple cycles of mechanical signals separated by periods of rest may promote osteogenesis and decrease fat formation [42]. Indeed, a vibrating belt (Osteoboost) to increase bone density has been recently approved by the Food and Drug Administration (FDA) of the United States of America [50].

Coupled with hypoxic culture conditions, mechanical stimulation could better recapitulate the tissue niche of bone-marrow MSCs. That way, they can be helpful to a more precise control and better understanding of factors affecting MSC differentiation [20]. The aim of this study is to highlight the potential of this strategy for the development of non-pharmacological therapies to help maintain or improve bone mass in frail patients with physical activity difficulties. To this purpose, we investigated the effects on the differentiation of human bone marrow-derived MSCs into osteoblasts or adipocytes of: (a) expansion of MSCs under cyclic low vibration stimuli in hypoxic conditions, followed by differentiation in normoxia (pre-condition or pre-treatment); and (b) induction of differentiation of MSCs into osteoblasts or adipocytes under cyclic low vibration stimuli in hypoxic conditions.

## 2. Materials and Methods

### 2.1. MSCs Culture and Differentiation

Human MSCs were isolated from commercial cryopreserved mononuclear cells from three donors (Stemcell Technologies, Cologne, Germany) according to the protocol previously optimized by our group [51]. The MSCs were seeded in 75 cm^2^ flasks from Nalgene-Nunc—Thermo Fisher Scientific (Waltham, MA, USA). They were grown in Minimum Essential-Medium Alpha (MEMα) from Biowest (Nuaillé, France), containing 2 mM UltraGlutamine (Biowest), 15% fetal bovine serum (FBS) (Gibco—Thermo Fisher Scientific), 100 U ampicillin, 0.1 mg streptomycin/mL, and 1 ng basic fibroblast growth factor (bFGF)/mL from Sigma-Aldrich (Saint Louis, MO, USA). Cultures were incubated at 37 °C with 95% humidity and 5% CO_2_. The medium was changed every three to four days.

Cells were detached with trypsin/EDTA (Biowest) when reaching near 90% confluence. Then, they were seeded in 12- and 24-well culture plates (Nalgene-Nunc—Thermo Fisher Scientific) with the same medium, but with 10% FBS, at a density of about 1000 cells/cm^2^. When cultures were near confluence, they were induced to differentiate into osteoblasts or adipocytes or remained undifferentiated. Osteoblastic differentiation was induced with 10 nM dexamethasone, 0.2 mM ascorbic acid, and 10 mM β-glycerolphosphate (Sigma-Aldrich) for 21 days. Differentiation into adipocytes was induced by 500 nM dexamethasone, 0.5 mM isobutylmethylxanthine, and 50 μM indomethacin (all from Sigma-Aldrich), being maintained for 14 days.

### 2.2. Experimental Conditions

#### 2.2.1. Pretreatment and Differentiation into Osteoblasts and Adipocytes

**Figure 1:**
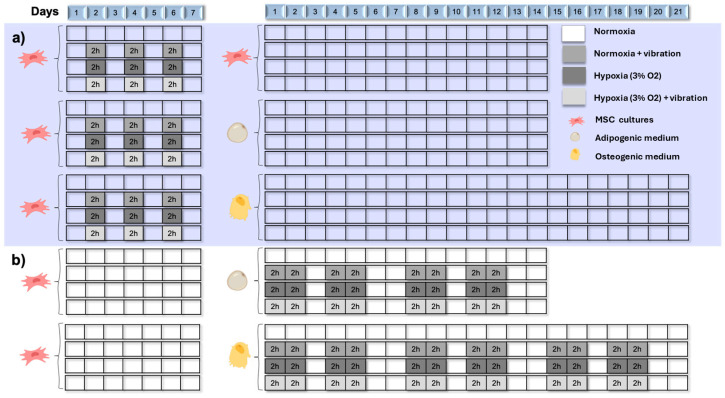
Temporal distribution of MSCs subjected to low-intensity vibration and cyclic hypoxia treatments. (**a**) Human MSCs were exposed to four different conditions during their expansion before confluence (seven days). Then, cultures were maintained in a conventional incubator during 14 (adipocytes and undifferentiated MSCs) or 21 (osteoblasts) days. (**b**) When MSCs were induced to differentiate into osteoblasts or adipocytes, tissue cultures were exposed to four different conditions during 14 (adipocytes) or 21 (osteoblast) days. Credit: illustration created with BioRender <https://www.biorender.com>.

Human MSCs were exposed to four different conditions during their expansion before reaching confluence and being induced to differentiate (Figure 1a). During seven days, these cultures were exposed to three sessions of two hours of duration of: normoxia (N), hypoxic cyclic (H), low-intensity vibration (NV), or combination of cyclic hypoxia and low-intensity vibration (HV). N and NV group cultures were maintained in standard culture conditions (5% CO_2_, 16% O_2_, 37 °C). Cultures of H and HV groups were exposed to 3% O_2_ during the hypoxia period.

Cultures of HV and NV groups were subjected to low-intensity vibration stimuli of 0.14 g and 12.8 Hz for the treatment period using a Multi-bio 3D mini-shaker from BioSan (Riga, Latvia). An OR36 dynamic signal analyzer from Oros (Grenoble, France) with a triaxial accelerometer was used to record the acceleration values, as a function of time, in all three axes. Of the recordings made, 10 s were analyzed with the signal regime remaining stable. A fast Fourier transform (FFT) was used to determine the root mean square (RMS) acceleration of each axis of the accelerometer in the main frequency domain. The resulting acceleration was determined as the vector sum of each of the three axes. After three sessions and seven days of culture, cells reached confluence, and cultures were differentiated into adipocytes or osteoblasts in standard culture conditions, as described above, without vibration stimuli.

#### 2.2.2. Cotreatments during Differentiation into Osteoblasts or Adipocytes

Cultures of MSCs induced to differentiate into osteoblasts or adipocytes were exposed to four different conditions (N, H, NV, and HV) during such differentiation (Figure 1b). Exposures were applied with a frequency of four days per week, during two hours per day, in different conditions (normoxia, hypoxia, and/or vibration), as described in the previous section.

### 2.3. Histochemical Stains

Alizarin-red staining at day 21 of osteoblastic differentiation was used for extracellular matrix mineralization visualization and quantification. In short, cultures in P12 plates were fixed with 3.7% formaldehyde for 10 min. Then, they were stained with 40 mM of alizarin red in water, adjusted to pH 4.15 with ammonium hydroxide (both chemicals from Sigma-Aldrich) for 10 min. Wells were then washed several times with 60% of isopropanol, dried, and visualized under light microscopy. Alizarin-red deposit measurements were carried out after elution with 10% acetic acid, neutralization with 10% ammonium hydroxide, and 405 nm spectrophotometric absorbance quantification of the resulting solution. The results were normalized with respect to the value obtained from the normoxia group.

Lipid accumulation in MSCs differentiated into adipocytes was determined by oil-red O staining. In short, cells were fixed and washed with isopropanol (60% in water) and stained for 15 to 20 min with a mixture of 60% of 0.3% oil red (*w*/*v* in isopropanol) and 40% of distilled water. After that, cells were washed twice in distilled water, stained with hematoxylin, and photographed under a light microscope. At least nine images were obtained from each well. The oil-red O stain in each image was quantified with the ImageJ software version 1.54f from the National Institutes of Health (NIH). Values were normalized with the number of cells per image. Lipid accumulation in cultures was expressed as oil-red O area per number of cells (area/cells).

### 2.4. RNA Isolation and Quantitative Real-Time Polymerase Chain Reaction (qRT-PCR)

RNA from culture cells was isolated using the NZY total RNA isolation kit from NZYTech (Lisbon, Portugal), following the manufacturer’s instructions. RNA was quantified with a NanoDrop ND-1000 Spectrophotometer (Thermo Fisher Scientific). Then, 900 ng were retrotranscribed with the iScript cDNA Synthesis Kit from Bio-Rad (Hercules, CA, USA).

Quantitative real-time PCR was carried out in a CFX96 Connect Instrument (Bio-Rad). Each reaction contained one μL of cDNA, 10 pmol of each primer pair (Table 1), and SensiFAST Sybr No-Rox Mix from Bioline (London, UK). The PCR amplification profile included one cycle at 95 °C for 2 min (DNA denaturation and hot-start DNA-polymerase activation) and 45 amplification cycles: 94 °C for 5 s (DNA denaturation) and 65 °C for 30 s (primer annealing and extension). Results were analyzed with CFX Maestro software V 2.3. (Bio-Rad). Polymerase (RNA; DNA-directed) II polypeptide A (*POLR2A*) and ribosomal protein L13a (*RPL13*) mRNA were used as housekeeping controls. Relative gene expression respect to the normoxia group was quantified using the 2^−ΔΔCt^ method, where Ct is the cycle threshold. 

### 2.5. Protein Extraction and Western-Blot Analyses

Cells were lysed with Cell Extraction Buffer (Thermo Fisher Scientific) supplemented with 1 mM of phenylmethylsulfonyl fluoride (PMSF) and 50 μL of protease inhibitor cocktail (PIC)/mL (both from Sigma-Aldrich), for total protein isolation. Lysates, once collected, were incubated in ice for 30 min, with vortex agitation every 10 min. Finally, they were centrifuged for 10 min (13,000× *g*) at 4 °C. Supernatants were transferred into new tubes and stored at −20 °C until used. Protein concentrations were quantified with the Bio-Rad DC Protein Assay kit (Bio-Rad), according to the manufacturer’s directions.

Subsequently, 10–20 μg of protein from each sample were loaded into 8 to 16% acrylamide nUView Tris-Glycine Precast gels from NuSeP (Germantown, MD, USA) under denaturing conditions. Electrophoresis was carried out in a Mini-Protean system (Bio-Rad). Then, proteins were transferred into polyvinylidene difluoride (PVDF) membranes (Bio-Rad) by a Trans-Blot Turbo Transfer System from the same manufacturer. Membranes were blocked with a 5% solution of skimmed milk in Tris-Tween Buffered Saline (TTBS) buffer (20 mM Tris-HCl pH 7.6, 150 mM NaCl, 0.05% Tween) for one hour at room temperature.

Then, membranes were incubated overnight at 4 °C with primary antibodies anti-β-catenin (1:1000), COL1A1 (1:1000), and α-tubulin (1:1000) from Cell Signaling Technology (Danvers, MA, USA), and β-actin (1:20,000) from Novus Biologicals (Centennial, CO, USA) in TTBS. After incubation, membranes were washed with TTBS and incubated for one hour with anti-rabbit (1:3000) or anti-mouse (1:1000) secondary antibody, conjugated to horseradish peroxidase (HRP), from Cell Signaling Technology in TTBS. Finally, membranes were developed with Clarity Western ECL Substrate and visualized in a ChemiDoc XRS+ gel documentation system. Acquisition and analyses of images were performed using Image Lab software version 6.0 (all three from Bio-Rad). For the normalization of the signals of the different proteins, stain-free technology was used. In short, gels or membranes were UV light activated in the ChemiDoc XRS+ Gel Imaging System (Bio-Rad), and images of total protein loaded for each sample were generated (Figures S1–S4). Total protein signals were quantified with Image Lab software version 6.0 [52,53], and the values obtained were used for normalization of the band intensity of evaluated proteins. Results are expressed as expression relative to the values of the control group (normoxia group).

### 2.6. Statistical Analyses

Statistical analyses were performed using the GraphPad Prism 6.0 program from GraphPad Software (San Diego, CA, USA). Standard statistical methods were used for the calculations of the mean and standard deviations. Experiments were carried out in triplicate, showing the average ± standard error of the mean (SEM). Analyses of variance (ANOVA) and Fisher’s projected least-significant difference (PLSD) tests were used to calculate *p* values. Differences were considered statistically significant when *p* < 0.05.

## 3. Results

### 3.1. Pretreatment with Low-Intensity Vibration Stimuli (Combined with Hypoxic Exposure) Predisposes MSCs Toward an Osteoblastic Phenotype

Human MSCs pretreated under N, NV, H, and HV conditions during three sessions, two hours per session, for seven days were subsequently maintained under standard culture conditions (5% CO_2_, 16% O_2_, 37 °C) for another 14 days. The putative effects of pretreatments on such cells, in relation to their predisposition to differentiate into osteoblasts or adipocytes, were studied. Thus, expression of adipogenic *PPARG2* and lipoprotein lipase (*LPL*), as well as osteogenic runt-related transcription factor 2 (*RUNX2*) and osterix (*SP7*) genes, encoding two transcription factors involved in the induction of differentiation into osteoblasts, was studied in these undifferentiated cells at 0, 7, and 14 days after exposure to the different treatments. The *PPARG2* gene encodes a key transcription factor for adipogenic differentiation, which activates transcription of adipogenic genes. Among them is *LPL*, which encodes a lipoprotein lipase involved in the formation of lipid vesicles. The expression of these two genes was not detected in any of the cultures subjected to the different treatments. This indicates that none of the treatments induced the cells towards an adipogenic phenotype.

**Figure 2:**
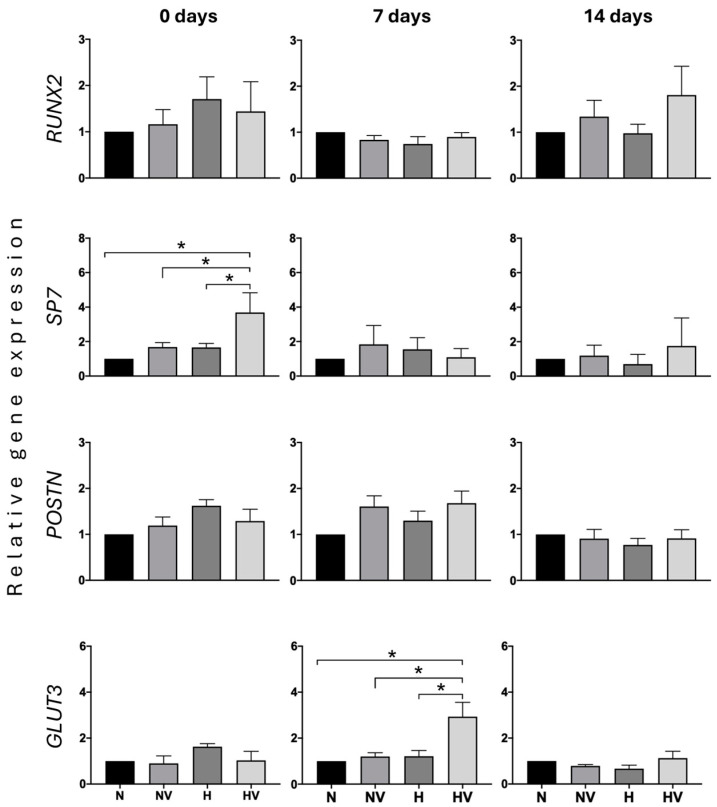
Quantification of gene expressions in MSCs pretreated with low-intensity vibration stimuli and/or hypoxia. The ones of osteoblastic gene markers (runt-related transcription factor 2 and SP7), periostin, and glucose transporter 3 were measured in MSCs after their expansion under different treatments: normoxia (N), cyclic hypoxia (H), low-intensity vibration (NV), and cyclic-hypoxia combined with low-intensity vibration (HV), the next day (0 d), 7 and 14 days after application of treatments. * *p* < 0.05.

However, the expression of the genes encoding the two osteogenic transcription factors (*RUNX2* and *SP7*) was expressed in all cultures at the three times studied (Figure 2). At time 0, *RUNX2* mRNA levels did not significantly change between treatments. However, *SP7* expression at this time was significantly increased in cultures previously treated with HV, with respect to other treatments, mainly with respect to cultures maintained in normoxia (Figure 2). This indicates that HV treatment predisposes cells to differentiate into osteoblasts. However, at 7 and 14 days after the cells were treated, no significant differences were observed in the expression of *RUNX2* and *SP7* among the different treatments. That suggests a loss of intensity of the stimuli effect with time (Figure 2).

In order to study a marker related to osteoblastogenesis and mechanical stimuli in MSCs after different treatments, the expression of the gene coding for periostin (*POSTN*) was evaluated. It is involved in cellular interactions within the extracellular matrix in the early differentiation of osteoblasts, among other functions. Moreover, its expression in bone is induced by mechanical stimuli and hypoxia [54,55]. In our study, at times 0 and 7 days, cells treated with vibration, with or without exposure to hypoxia, and those treated only with hypoxia showed slightly higher POSTN mRNA levels than cultures treated with normoxia, but not statistically significant. Therefore, these stimuli cannot be considered to have the capacity to affect the expression of this gene (Figure 2).

Also, as a marker of the possible metabolic changes produced by treatments, expression of the gene encoding glucose transporter 3 (*GLUT3*) was analyzed. That is one of the main glucose transporters in osteoblasts, whose expression is regulated by hypoxia [30,56]. *GLUT3* increased its expression after the end of treatments (T = 0) in hypoxia-treated cultures, albeit such changes were not statistically significant. However, at day 7 after the application of the stimuli, HV-treated cells showed an induction of gene expression, which was not maintained at day 14 (Figure 2). Therefore, the increased expression of *GLUT3*, *POSTN,* and *SP7* genes in undifferentiated MSCs pretreated with HV suggests that such treatment predisposes these cells to differentiate into osteoblasts.

### 3.2. Effect of Pretreatment with Low-Intensity Vibration and/or Cyclic Hypoxia on MSCs Differentiation into Osteoblasts or Adipocytes

Human MSCs were pretreated during expansion for 7 days with three two-hour sessions, using low-intensity vibration, hypoxia, or a combination of both, as described in the material and methods. In order to evaluate whether the different treatments could influence the differentiation capacity of the cells, pretreated MSCs were subsequently induced to differentiate into osteoblasts or adipocytes under standard culture conditions.

**Figure 3:**
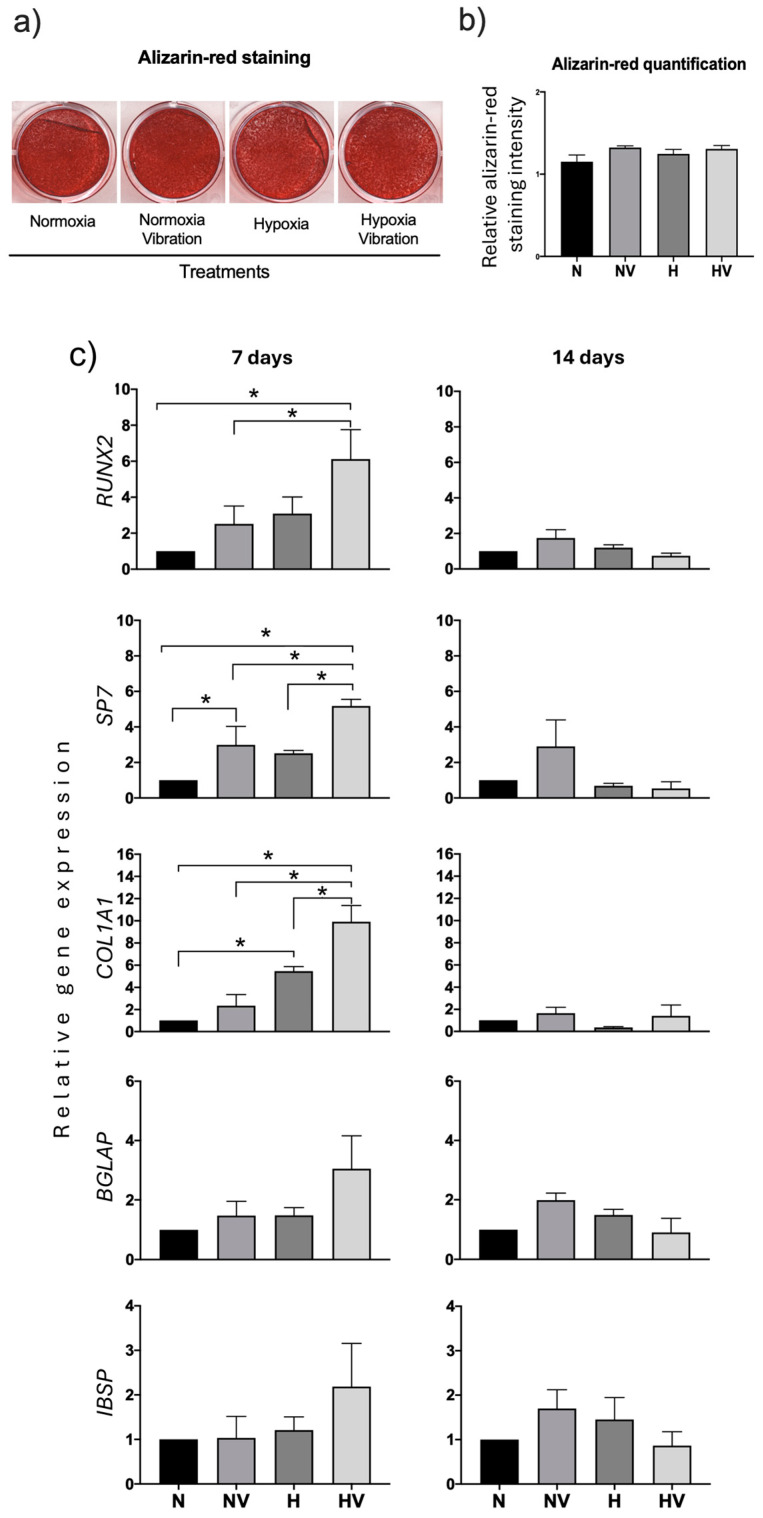
Effects of pretreatment of cyclic hypoxia and low-intensity vibration on MSCs induced to differentiate into osteoblasts. MSCs were expanded and induced to differentiate into osteoblasts under different conditions. See legend of Figure 2. The ones of osteoblastic markers (runt-related transcription factor 2, SP7, collagen type I alpha 1, osteocalcin, and integrin-binding sialoprotein) were measured at days 7 and 14 after being induced to differentiate into osteoblasts. At day 21, the extracellular matrix mineralization of the cultures was stained with alizarin red. (**a**) Representative images of alizarin-red staining of cultures differentiating into osteoblasts under the different conditions; (**b**) Alizarin-red quantification; (**c**) Quantification of osteoblastic-marker gene expressions in induced osteoblasts of preconditioned MSCs. * *p* < 0.05.

In MSCs differentiated into osteoblasts, no differences were observed at day 21 of differentiation between the different treatments in relation to mineralization of the extracellular matrix (Figure 3a,b). However, the study of the expression of osteoblastic marker genes showed that the genes encoding transcription factors *RUNX2* and *SP7* were induced in cultures previously subjected to vibration at day 7 of differentiation. In the case of *SP7*, both under hypoxia and normoxia conditions. The expression of genes encoding proteins related to the extracellular matrix, such as collagen type I (*COL1A1*), bone sialoprotein (*IBSP*), and osteocalcin (*BGLAP*), was also studied. At 7 days of osteogenic differentiation, only *COL1A1* gel expression was significantly increased in cultures exposed to hypoxia with or without vibration. At 14 days, no significant changes were observed in any of the osteogenic genes studied in response to the different pretreatments performed (Figure 3c).

**Figure 4:**
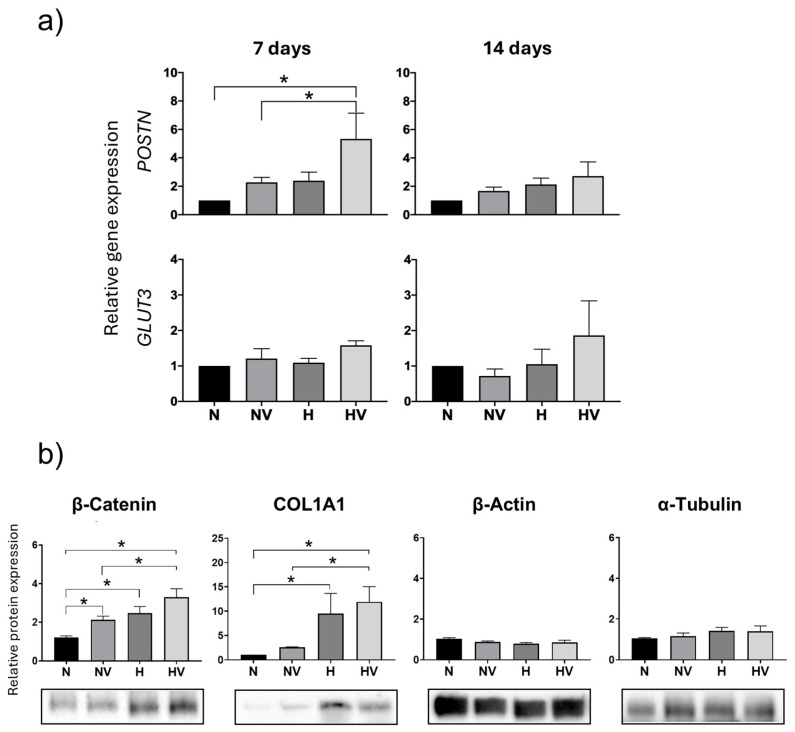
Quantification of gene expressions and protein synthesis in MSCs pretreated and then induced to differentiate into osteoblasts. (**a**) Periostin and glucose transporter 3 were measured at days 7 and 14, and (**b**) β-catenin, COL1A1, β-actin, and α-tubulin were determined by Western blot at day 14 in induced osteoblasts from mesenchymal-stem cells after their expansion under different treatments. See legend of Figure 2. * *p* < 0.05.

In cultures differentiated into osteoblasts, the expression of *POSTN* and *GLUT*3 was also studied. *POSTN* expression increased at 7 days in the cultures pretreated with hypoxia plus vibration compared to the groups exposed to normoxia. While no significant differences were observed in the expression of GLUT3 at any of the times studied (Figure 4a). 

On the other hand, beta-catenin is one of the main pathways involved in MSC differentiation. Its activation induces the differentiation of cells towards osteoblasts, whereas its inhibition is necessary for adipogenic differentiation. In cultures differentiated into osteoblasts, after pretreatment with vibration and/or hypoxia, beta-catenin protein levels were studied at day 14 after differentiation. The results showed that both vibration and hypoxia increased beta-catenin synthesis. In addition, COL1A1 protein levels were also increased in HV- and H-pretreated cultures compared to those maintained in normoxia. In both proteins, the greatest increase occurred when both stimuli were combined (Figure 4b). This can be correlated with what was observed for osteoblast-marker gene expression in cultures pretreated with vibration hypoxia.

In addition, in order to study whether hypoxia and/or mechanical stimulation could produce any effect on the expression of proteins related to the cytoskeleton, the expression of beta-actin and alpha-tubulin was studied at day 14 of osteogenic differentiation. Statistically significant changes were not observed in any of such proteins between the different treatments (Figure 4b). 

**Figure 5:**
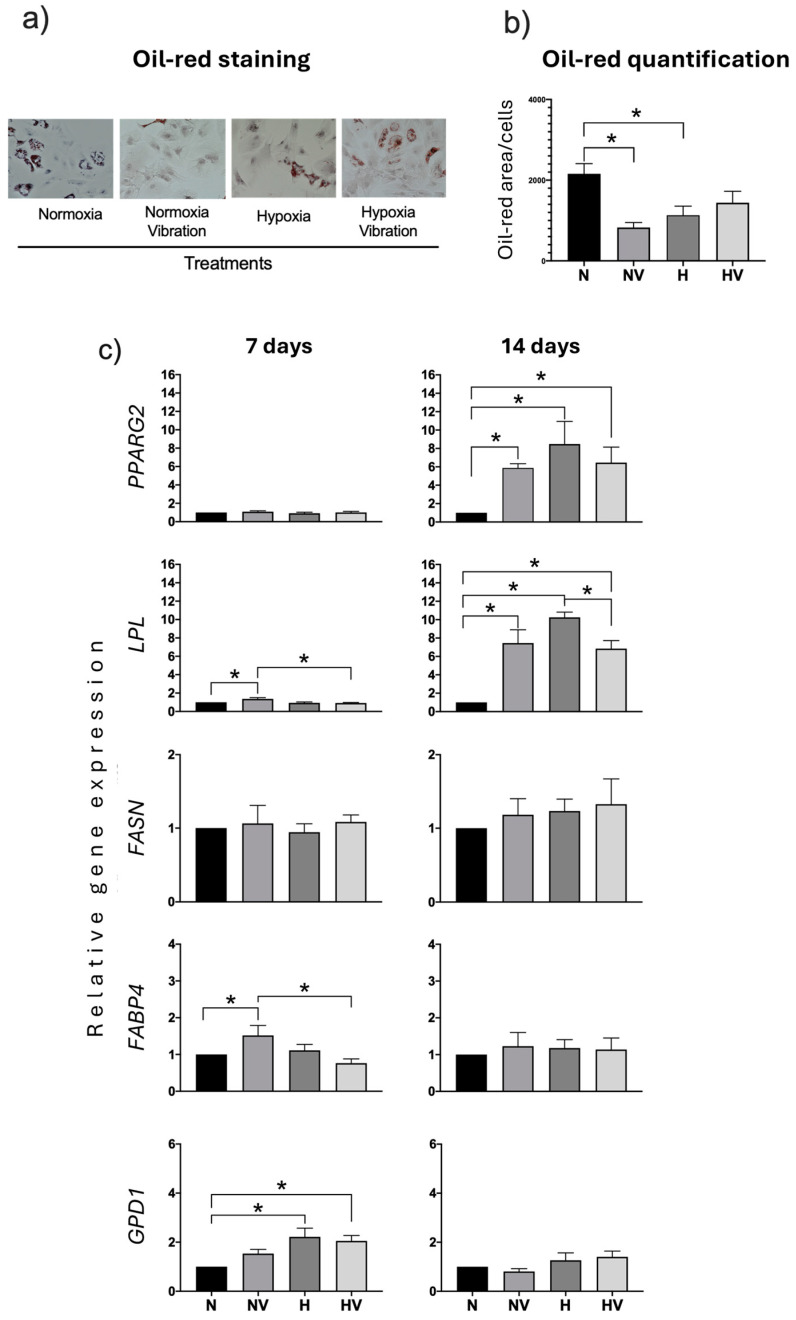
Effects of pretreatment of cyclic hypoxia and low-intensity vibration on MSCs and induced differentiation into adipocytes. MSCs were pretreated under different conditions and induced to differentiate into adipocytes. Genes encoding adipogenic markers (peroxisome proliferator-activated receptor gamma 2, lipoprotein lipid, fatty acid synthase, fatty acid-binding protein 4, and glycerol-3-phosphate dehydrogenase 1) were measured at days 7 and 14 after being induced to differentiate into adipocytes. Cultures were stained with oil-red O to reveal lipid droplets at day 14. (**a**) Representative images of oil-red O staining of cultures differentiating into adipocytes under the different conditions; (**b**) Oil-red quantification; (**c**) Quantification of adipogenic-marker gene expressions in induced adipocytes of preconditioned MSCs. * *p* < 0.05.

With respect to adipogenic differentiation, the results showed that exposure of MSCs to low vibration and/or hypoxia, before initiating differentiation, decreased the formation of fat vesicles in pretreated cells, compared to those maintained in normoxia at 14 days of differentiation, as observed after staining with oil-red O (Figure 5a,b). The decrease was significant in cells pretreated with NV and H. Interestingly, analyses of mRNA levels of the *PPARG2* gene, which is a key transcription factor for adipogenic differentiation, showed that, at day 7 of differentiation, there was no difference between different treatments. However, at day 14, cells pretreated in NV, H, or HV presented higher values than those maintained in normoxia. The same was observed for the gene encoding LPL (Figure 5c). The study of other adipocytic-marker genes related to lipid metabolism, such as fattyacid synthase (*FASN*), fatty acid-binding protein 4 (*FABP4*), and glycerol-3-phosphate dehydrogenase 1 (*GPD1*), showed that different pretreatments did not produce variations in mRNA levels at day 14. However, at day 7, an increase of *FABP4* was observed in cultures exposed to NV and of *GPD1* in cultures exposed to hypoxia (H and HV) (Figure 5c).

**Figure 6:**
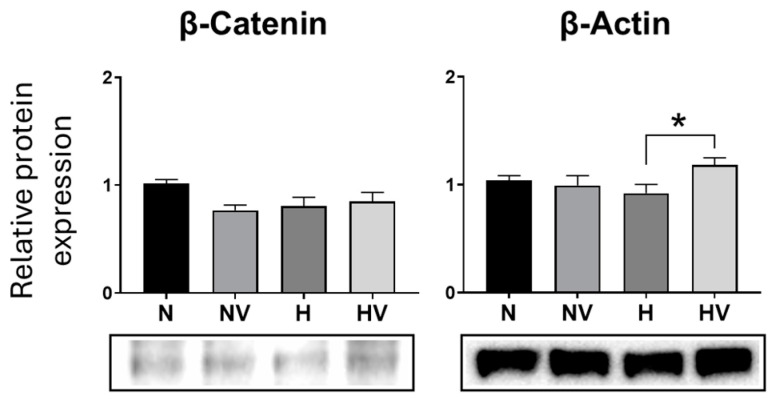
Effects of the combination of cyclic hypoxia and low-intensity vibration pretreatment on protein synthesis in MSCs differentiated into adipocytes. β-catenin and β-actin were quantified by Western blot at day 14, after being induced to differentiate into adipocytes. * *p* < 0.05.

Quantification of beta-catenin protein at day 14 of adipogenic differentiation showed that pretreatments did not produce statistically significant changes (Figure 6). In addition, changes were not observed for the synthesis of beta-actin protein in relation to cultures in normoxia. Although cultures pretreated with a combination of hypoxia and vibration did show an increase in the synthesis of beta-actin with respect to pretreatment with hypoxia only (Figure 6).

### 3.3. Effects of Cotreatment with Low-Intensity Vibration and/or Cyclic Hypoxia in MSCs Differentiation into Osteoblasts or Adipocytes

The putative effect on MSC cell differentiation of low-intensity vibration, hypoxia, or a combination of both was studied. Thus, cells differentiating into osteoblasts or adipocytes were exposed to four sessions per week, of two hours each, to different treatments, as described in material and methods.

**Figure 7:**
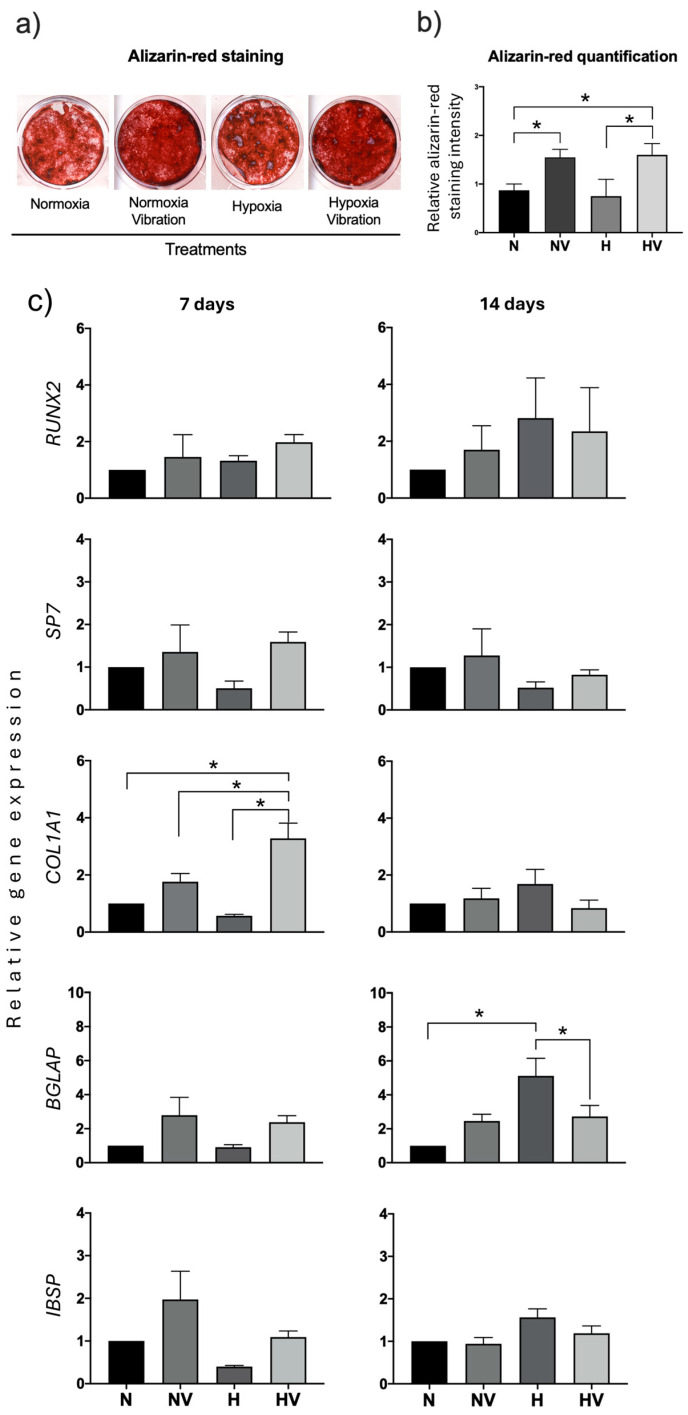
Effects of a cotreatment of cyclic hypoxia and low-intensity vibration on differentiation of induced osteoblasts from MSCs. Mesenchymal stem cells were induced to differentiate into osteoblasts under different conditions, 4 days per week. Osteoblastic markers were measured at days 7 and 14. At day 21, the extracellular matrix mineralization of the cultures was stained with alizarin red. (**a**) Representative images of alizarin-red staining of cultures differentiating into osteoblasts under the different conditions; (**b**) Alizarin-red quantification; (**c**) Quantification of osteoblastic-marker gene expressions in induced osteoblasts of MSCs under different conditions. * *p* < 0.05.

Human MSC cultures differentiating into osteoblasts and exposed to cyclic hypoxia and vibration at day 21 showed greater mineralization of the extracellular matrix compared to cultures treated only with cyclic hypoxia or maintained in normoxia. Exposure to vibration of cultures maintained in normoxia also produced an increase in mineralization (Figure 7a,b). These data suggest a positive effect of vibration on the mineralization of the extracellular matrix in osteoblasts.

The study of the expression of genes encoding transcription factors RUNX2 and SP7 showed that treatments did not produce statistically significant changes (Figure 7c). However, with HV treatment, at day 7, COL1A1 gene expression increased in relation to all other treatments. At day 14 of differentiation, the most remarkable result was the increased BGLAP gene expression in cultures exposed to hypoxia (Figure 7c). Therefore, the results suggest that different treatments evaluated may affect components of the extracellular matrix.

**Figure 8:**
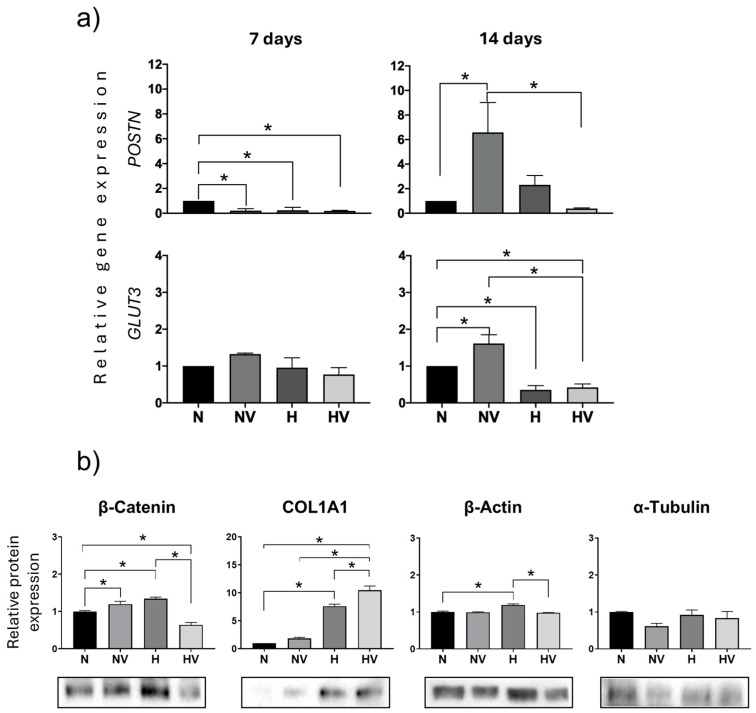
Quantification of gene expression and protein synthesis in MSCs differentiated into osteoblasts under different treatments of cyclic hypoxia and low-intensity vibration. (**a**) Periostin and glucose transporter 3 gene expression were measured at days 7 and 14; (**b**) β-catenin, COL1A1, β-actin, and α-tubulin were quantified by Western blot at day 14. * *p* < 0.05.

In these cultures, the expression of POSTN and GLUT3 genes was also studied at days 7 and 14 of differentiation. POSTN was repressed by all treatments at day 7. However, at a more advanced stage of differentiation (day 14), exposure to low-intensity vibration in cultures maintained in normoxia induced its expression. Regarding GLUT3 expression, it increased at both times studied in cultures treated with NV, mainly at day 14 of differentiation, with respect to cultures exposed to hypoxia with or without vibration (Figure 8a). Beta-catenin expression at day 14 of osteogenic differentiation increased in cultures exposed to vibration or hypoxia but not in cultures exposed to both stimuli. A decrease was observed in the latter. However, protein expression of COL1A1 increased in hypoxia groups, mainly in those combined with vibrational stimulus (Figure 8b). This correlates with the expression changes observed for *the* COL1A1 gene. In relation to beta-actin and alpha-tubulin protein synthesis, as marker proteins of the cytoskeleton, an increase in beta-actin levels was only observed in cultures exposed to cyclic hypoxia during differentiation into osteoblasts (Figure 8b).

**Figure 9:**
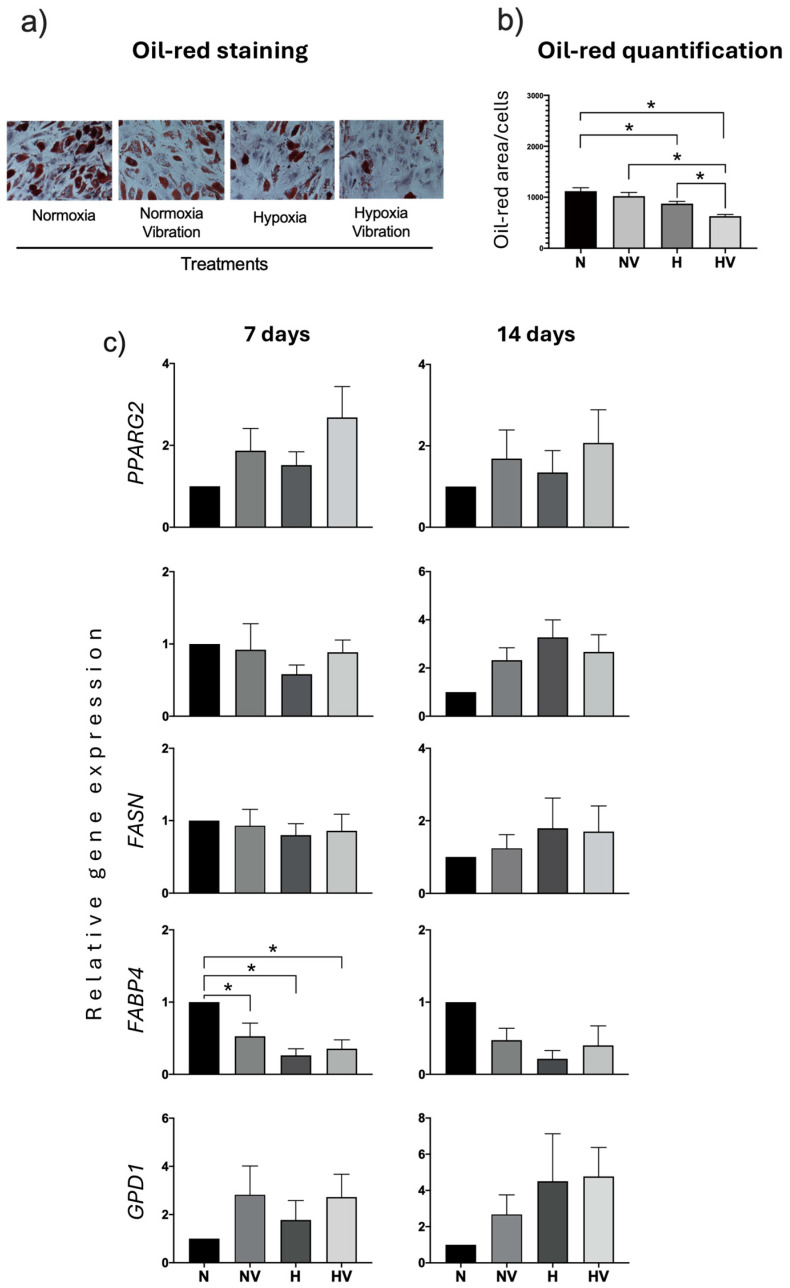
Effects of cyclic hypoxia and low-intensity vibration on MSCs differentiated into adipocytes under different conditions. Genes encoding adipogenic markers were measured at days 7 and 14. Cultures were stained with oil-red O to reveal lipid droplets at day 14. (**a**) Representative images of oil-red O staining of cultures differentiating into adipocytes under the different conditions; (**b**) Oil-red quantification; (**c**) Quantification of adipogenic-markers gene expressions in induced adipocytes from MSCs. * *p* < 0.05.

In MSCs exposed to cyclic hypoxia and/or low-intensity vibration during the induction of adipogenic differentiation, lipid droplet formation was decreased in all cases, mainly in cultures exposed to cyclic hypoxia or cyclic hypoxia in combination with low-intensity vibration (Figure 9a,b). The expression of adipogenic genes, such as *PPARG2*, *LPL*, *FASN,* and *GPD1*, did not show significant differences between different treatments (Figure 9c). 

**Figure 10:**
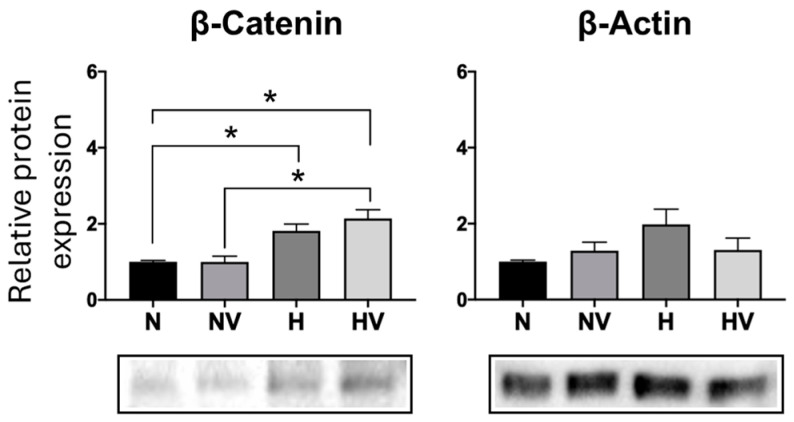
Effects of combination of cyclic hypoxia and low-intensity vibrations on protein synthesis in MSCs differentiated into adipocytes. β-catenin and β-actin were determined by Western blot at day 14 under different conditions. * *p* < 0.05.

However, the expression of the *FABP4* gene decreased at day 7 of differentiation with all treatments, mainly those using cyclic hypoxia (Figure 9c). These changes in adipogenic gene expression were accompanied by increased protein synthesis of beta-catenin in cultures treated with cyclic hypoxia alone or in combination with vibration (Figure 10). Cytoskeleton-related protein beta-actin showed no significant differences between different treatments (Figure 10).

## 4. Discussion

The main finding of this study has been to demonstrate that combination of cyclic hypoxia stimuli with low-intensity vibration favors osteogenic MSC differentiation, inhibiting adipogenic differentiation. Due to the positive effect of this strategy on osteoblast precursor cells, it may have a high clinical potential for use in bone regeneration therapies, mainly in frail patients with significant mobility limitations. 

When MSCs were pretreated with three sessions (two hours each) of low-intensity vibration in combination with hypoxia, our results showed increased expression of the gene encoding the SP7 osteoblastic transcription factor in MSCs not induced to differentiate. These results are in agreement with the scientific literature. Thus, MSCs preconditioning under hypoxic conditions can lead to activation of multipotency and ability to differentiate. Interestingly, a combination of hypoxia with mechanical stimuli can recapitulate or simulate the physiological conditions of MSC niches in bone marrow [20]. Mechanical signals have been interpreted as stimulatory for osteogenesis and inhibitory for adipogenesis [57]. Moreover, the adaptations observed in HV-preconditioned cultures could be mediated by the synergic effects of mechanical stress and hypoxic exposure.

Increased predisposition towards osteoblastic differentiation could be favored by increasing glycolytic metabolic pathways. That was shown by increased *GLUT3* gene expression in these cultures compared to other conditions. Such an increase in expression occurred 7 days after the end of stimuli and the induction of *SP7*. Therefore, it is likely related to a greater commitment of these cells to differentiate into osteoblasts. It should be considered that *GLUT3* is one of the main glucose transporters in osteoblasts [58]. Additionally, energy production is mainly mediated through the Warburg effect in these cells, generating ATP from glycolysis, which is aerobic [59]. However, 14 days after the end of the treatments, no differences were observed in the expression of osteoblastic genes between different treatments. The rationale is that the effects observed in MSCs subjected to hypoxia and vibration diminished with time due to a lack of stimuli.

This idea is also supported by results obtained in MSCs differentiating into osteoblasts after being preconditioned with several sessions of low-intensity vibration in the presence or absence of hypoxia. In such cultures, no differences of extracellular matrix mineralization were observed, comparing cultures pretreated with different stimuli after 21 days of osteogenic differentiation. However, studies of gene expression and protein synthesis showed that HV pretreatment induced expression of osteoblastic markers in early stages of differentiation. These results are in agreement with those described in recent studies. Thus, it has been shown that MSCs preconditioning under hypoxia, followed by differentiation under normoxia, can increase the differentiation potential compared to differentiation under normoxia or hypoxia alone [20].

Upregulated osteoblastic-marker gene expressions (*RUNX2*, *SP7*, and *COL1A1*) in preosteoblasts were shown after HV preconditioning. However, no change was observed at day 14 versus cultures maintained in normoxia. Equal differentiation potential of MSCs after preconditioning with both normoxia and hypoxia has also been previously reported [21,27,60]. Gene expression of *POSTN* and *GLUT3* further supports this argument. This way, the expression of *POSTN* significantly increased at day 7. The glucose metabolism of undifferentiated MSCs is mainly based on glycolysis. In addition, it is induced by HIF1 after hypoxic stimuli. However, subsequently, oxidative phosphorylation must be activated during mineralization, allowing maturation of preosteoblasts into osteoblasts [61]. In addition, *POSTN* acts as a ligand, binding to heterodimeric integrin receptors. They include integrin-α6β4, being relevant in survival and differentiation of bone MSCs [54]. Its expression has been identified in MSCs, being considered a specific marker for preosteoblasts [62]. This matrix protein can activate osteogenesis of osteoblast precursors through integrin receptors and the WNT-β-catenin signaling pathway. Indeed, that is one of the main signaling pathways responsible for osteogenic differentiation [63,64].

In relation to that, β-catenin protein increased at day 14 after HV preconditioning. For all that, our results agree with the hypothesis that preconditioning under vibration and hypoxia predisposes MSCs towards an osteoblastic lineage. However, does not have the ability to increase mineralization of the extracellular matrix, although a higher expression of COL1A1 is shown in these cultures. This suggests that such stimuli are not sufficient to promote the induction of osteoblastic maturation. Various factors could be influencing. These may include time of exposure to hypoxia and/or vibration, O_2_ concentration, substrates on which cells are cultured, growth factors used to induce differentiation, and other experimental procedures. In this sense, previous research has shown how two-dimensional (2D) versus three-dimensional (3D) culture conditions could influence the behavior and differentiation of MSCs [65]. Some previous studies have used 3D models to confirm the positive effects of vibrational stimulation on osteogenic differentiation in the same way as in 2D cultures [66]. Further research may therefore be needed to understand the effects of this novel combined stimulus on 3D cultures using different matrix types, which would more closely resemble the in vivo situation.

It has been established that different mechanosensitive pathways can modulate hypoxia-influenced factors [20]. In this respect, several authors have reported that low-intensity vibration had anabolic effects on bone, suppressing adipose tissue formation [42]. Factor dynamics (duration of treatment) of the load environment (cycle number, strain rate, and impact strength intensity) are more strongly correlated with additional influences than to load magnitude. Therefore, incorporation of multiple cycles of mechanical signals, separated by periods of rest (14 s to 8 h between mechanical inputs), can increase osteogenesis, reducing adipogenesis [67]. Thus, the specific details of the exposure procedure to hypoxia and vibration of MSCs could differentially modulate osteogenic differentiation. As said before, MSC preconditioning under hypoxic conditions can lead to upregulation of multipotency, being a viable strategy to enhance the differentiation potential of MSCs. Yet, optimization of the stimuli methodology may be of great relevance to ensure a minimal effective dose, effectively achieving desired changes, to enhance extracellular matrix mineralization.

The intensity or maintenance of low vibration stimuli in conditions of normoxia or hypoxia in the osteoblastic differentiation of MSCs is relevant. That is evidenced by results obtained when exposure to different treatments is carried out during osteogenic differentiation. In this case, contrary to pretreatment, greater mineralization is observed in cultures exposed to low-intensity vibration. That was observed under both normoxic conditions and cyclic hypoxia, with respect to cultures maintained in normoxia without vibratory stimuli. In cultures treated with HV, expression of the *COL1A1* osteoblastic marker gene, as well as the protein expression of COL1A1, were also increased. This indicates that vibratory stimuli are the main responsible for the observed osteogenic induction. In addition, these cultures showed higher values than those maintained in normoxia under vibratory stimuli, suggesting also that hypoxia may affect the response of MSCs during osteoblastic differentiation when intermittently treated with vibratory stimuli.

Indeed, at day 14 of osteoblastic differentiation, mRNA levels of genes encoding periostin and GLUT3 glucose transporter were different between NV and HV treatments. Interestingly, mRNA levels of the *POSTN* gene in all cultures exposed to vibration and/or hypoxia sessions were lower than when maintained in normoxia at day 7 of differentiation. It should be noted that periostin has been identified as a preosteoblastic marker, with its expression mainly increasing at the start of osteogenic differentiation [68]. Therefore, our results could indicate that the treatments are negatively affecting MSC differentiation into osteoblasts. However, expression of genes encoding RUNX2 and SP7 osteoblastic transcription factors in the cultures treated at that time should be considered. Thus, they did not show statistically significant changes in expression with respect to the cultures maintained in normoxia. Therefore, the observed decrease in *POSTN* mRNA levels at day 7 of differentiation in cultures exposed to NV, H, or HV may be related to other factors. They may include cellular adaptative responses to changes in the microenvironment. Among them are the ones produced by cyclic treatments of exposure to low-intensity vibration and hypoxia stimuli. These are some stresses that may significantly affect *POSTN* transcription [24,55].

Thus, in a mouse model of occlusal hypofunction, a decrease in periostin gene mRNA levels was observed in the periodontal ligament at 12, 24, and 72 h after tooth extraction. These were recovered 168 h (7 days) after intervention [69]. Another interesting result regarding *POSTN* gene expression was obtained in the present study. Thus, at day 14 of differentiation, it was significantly increased in cultures treated only with vibrational stimulation (NV). That was not observed in cultures additionally exposed to hypoxia (HV). This suggests that cyclic hypoxia counteracted the inducing effect of vibration on *POSTN* expression at that time of differentiation. This may be related to the fact that both hypoxia and mechanical stimuli can affect gene expression, albeit through different pathways. For example, hypoxia can upregulate the Yin Yang 1 (YY1) transcription factor [70], which is a repressor of *POSTN* transcription [71]. However, the differences observed between NV and HV exposure did not significantly affect extracellular matrix mineralization at day 21 of differentiation. Nevertheless, HV-exposed cultures showed lower beta-catenin protein synthesis at day 14. Therefore, that suggests that this treatment accelerates osteoblast maturation. The rationale is that in the later stages of osteoblast differentiation, beta-catenin levels must decrease to allow osteoblastic maturation [72]. In other words, decreased levels of *POSTN* mRNA and beta-catenin protein at day 14 in HV-exposed cultures may be related. This hypothesis is also supported by the fact that lower *GLUT3* gene expression was observed in these cultures. Bone cells require stable *GLUT3* expression to ensure basal glucose uptake. However, when faced with high-energy demands (such as the combination of stimuli), changes in cellular distribution of *GLUT3* have been observed in different cell models. They include osteoblasts, even when transcription of the gene is repressed [58,73]. Moreover, in the process of differentiation from precursor osteoblasts to mature osteoblasts, an interesting process has been described. Curiously, there is a shift from mainly glycolysis-based metabolism to increased oxidative phosphorylation [61,74]. That may affect glucose transport. Therefore, our study suggests that exposure of MSCs during osteogenic differentiation to low-intensity vibrational cycling under hypoxic conditions accelerates osteogenic differentiation and enhances extracellular matrix mineralization.

Regarding adipogenic differentiation, preconditioning of MSCs under hypoxia and/or vibration, followed by induction-adipogenic differentiation in normoxia conditions, decreased lipid-droplet formation. That happened with all pretreatments, mainly with hypoxia and vibration alone. However, early-stage *PPARG2* and *LPL*, which are adipogenic marker genes, showed upregulation in mature adipocytes. That was found at day 14 after differentiation started, compared with normoxia controls. Some authors have described that, in bone marrow-derived MSCs induced to differentiate into adipocytes, *PPARG2* expression increased during the first days of differentiation, with the appearance of preadipocytes. That decreased at day 14, with the presence of mature adipocytes [75]. Therefore, our results suggest that preconditioning MSCs with three cycles of hypoxia exposure, with or without low-intensity vibration, maintained MSCs in an undifferentiated or preadipocyte state. That was extended for a longer time, when cells were subsequently treated with adipogenic medium. This may explain why, at day 14, the increase in *PPARG2* expression did not lead to an increase in expression of genes involved in fat metabolism, such as *FASN* and *FABP4*. Consequently, cells showed a lower number of fat vesicles.

Our results also showed that, in cultures preconditioned with hypoxia or hypoxia plus vibration, at day 7 of adipogenic differentiation, expression of the gene coding for glycerol-3-phosphate dehydrogenase 1 (*GPD1*) increased. This enzyme catalyzes the reversible conversion of glycerol-3-phosphate to dihydroxyacetone phosphate. Both substrates can be used in different metabolic pathways, including glycolysis and lipogenesis, to form triglycerides. Overexpression of this gene in mice produced alterations in fat deposits, increasing brown fat and reducing white fat [76]. Recently, it has been described that, in renal clear cell carcinoma, hypoxia induced *GPD1* expression and decreased lipid production. That was probably due to glycerol 3-phosphate being shunted to glycerophospholipid production [77]. Our results suggest that preconditioning in hypoxia might have induced a similar effect. Indeed, hypoxia-pretreated MSC cultures exhibited increased *GPD1* transcription at day 7, after differentiation and exposure to treatments. Yet, at day 14, such an increase was not observed, and formation of fatty vesicles was lower compared to control cultures.

When stimuli were applied during the induction to differentiate MSCs into adipocytes, a statistically significant decrease in lipid-droplet formation was shown. That was observed in cultures treated with cyclic hypoxia or a combination of hypoxia and vibration, compared to the rest of the conditions. In the analyses of gene expression, *FABP4* was decreased, compared to the normoxia group, in all groups at day 7. This lower expression was maintained in mature adipocytes, being accentuated in the group subjected to hypoxia. FABP4 is mainly involved in trafficking of non-esterified fatty acids into cells, being also involved in storage of fatty acids as triacylglycerols. It is mainly expressed in adipocytes, where it is considered a marker of adipogenic differentiation. However, interestingly, it is also abundant in monocytes and macrophages. It is currently considered an adipokine, being associated with different pathologies such as obesity, inflammation, insulin resistance, cancer, and cardiovascular disease [78]. Although FABP4 is considered a marker of mature adipocytes, expression of its encoding gene has also been detected in preadipocytes. Inhibition of its expression in such cells decreased their capacity for adipogenic differentiation [79].

Finally, in cultures exposed to H and HV, an increase in protein levels of β-catenin compared to cultures maintained in normoxia was observed. Adipocyte formation decreased under hypoxic conditions by downregulation of adipogenic markers, such as *FABP4* and *LPL* [21,25,80]. Furthermore, application of mechanical stimuli in vitro inhibited adipogenesis by increasing beta-catenin signaling [81,82]. Synthesis of β-catenin counteracted and inhibited adipogenesis of bone marrow-derived MSCs. That was demonstrated by reduced lipid levels [82,83,84]. As described in the previous section, propagation of mechanical signals along the WNT-β-catenin pathway improved osteogenesis while inhibiting adipogenesis [49]. The application of physical forces in vitro inhibited adipogenesis and preserved the multipotentiality of MSCs, including their ability to enter the osteoblast lineage [85]. The combination of hypoxia and mechanic stimuli promotes effects on bone remodeling while reducing adiposity in the bone marrow.

## 5. Conclusions

Previous results from our group have shown that exposure to short periods of cyclic hypoxia can improve bone metabolism in the elderly. That was probably accomplished through reduction of bone resorption [31]. In the present study, we demonstrate that this strategy can be complemented with the combination of low-intensity vibratory stimuli to enhance the effect on bone formation and, therefore, its potential use in human clinical practice. This is due to the fact that the combination of both cyclic hypoxia and low-intensity vibrational stimuli has a greater capacity to favor osteoblastic differentiation, decreasing adipogenesis of bone marrow-derived MSCs. These effects were greater when stimuli occurred during the differentiation process. Nevertheless, when applied to undifferentiated cells, they also increased cell commitment to differentiate into osteoblasts instead of into adipocytes. Our gene expression and protein-synthesis studies showed that cyclic interventions modulated different physiological activities. Among them were expression of osteoblastogenesis-promoting genes, such as *RUNX2* and *SP7*; mineralization of extracellular matrices; expression of genes encoding proteins involved in adipocyte lipid formation and glucose metabolism; and synthesis of beta-catenin protein. However, more detailed studies are necessary to better understand the molecular mechanisms and signaling pathways involved in these phenomena. That should shed lighter on the effects of exposure to cyclic hypoxia, in combination with low intensity vibration, on the differential differentiation of MSCs into osteoblasts or adipocytes.

At the clinical level, our results have a high practical potential. Thus, they suggest the possibility of improving bone metabolism through exposure to normobaric cyclic hypoxia in combination with low-density vibratory stimuli. That is particularly relevant in elderly and/or frail people with difficulty performing physical activity. This is important, considering that this type of patient has a high risk of osteoporotic bone fractures, associated with both age and physical inactivity [86]. It should be noted that exposure to high-frequency and high-intensity vibration is not recommended to improve their muscular and skeletal systems. In addition, our results can also be considered for its application in cell therapy, aimed at the treatment of bone pathologies. In this case, it would be interesting to consider preconditioning MSC cultures under the conditions described in our study. That way, their osteogenic potential could be increased, being subsequently used in therapies for induction of bone formation.

## Figures and Tables

**Table 1:** Oligonucleotide primer pair sequences.

Gene	Primer Sequence (5′ → 3′) (Forward above; Reverse below)	Product Size (bp)
Runt-related transcription factor 2 (*RUNX2*)	TGGTTAATCTCCGCAGGTCAC ACTGTGCTGAAGAGGCTGTTTG	143
Osterix (*SP7*)	AGCCAGAAGCTGTGAAACCTC AGCTGCAAGCTCTCCATAACC	163
Collagen, type I, alpha 1 (*COL1A1*)	CGCTGGCCCCAAAGGATCTCCTG GGGGTCCGGGAACACCTCGCTC	263
Integrin-binding sialoprotein (*IBSP*)	AGGGCAGTAGTGACTCATCCG CGTCCTCTCCATAGCCCAGTGTTG	171
Osteocalcin (*BGLAP*)	CCATGAGAGCCCTCACACTCC GGTCAGCCAACTCGTCACAGTC	258
Periostin (*POSTN*)	CCAAATGTCTGTGCCCTTCAAC AGCCTTTCATTCCTTCCATTCTC	154
Transporter Glucose 3 (*GLUT3*)	AAACTTGCTGCTGAGAAGGACA AGAGCCGATTGTAGCAACTGTG	167
Peroxisome proliferator-activated receptor gamma 2 (*PPARG2*)	GCGATTCCTTCACTGATACACTG GAGTGGGAGTGGTCTTCCATTAC	136
Lipoprotein lipase (*LPL*)	AAGAAGCAGCAAAATGTACCTGAAG CCTGATTGGTATGGGTTTCACTC	113
Fatty acid synthase (*FASN*)	AAGCTGAAGGACCTGTCTAGG CGGAGTGAATCTGGGTTGATG	146
Fatty acid binding protein 4 (*FABP4*)	GCAGCTTCCTTCTCACCTTGA CCATGCCAGCCACTTTCCT	155
Glycerol-3-Phosphate Dehydrogenase 1 (*GPD1*)	ATACAGCATCCTCCAGCACAAG GGATGATTCTGCAGGCAGTG	120
Polymerase RNA II polypeptide A (*POLR2A*)	TTTTGGTGACGACTTGAACTGC CCATCTTGTCCACCACCTCTTC	125
Ribosomal Protein L13a (*RPL13a*)	CTCTGGACCGTCTCAAGGTGT CTGGTACTTCCAGCCAACCTC	158

## Data Availability

Dataset available on request from the authors.

## Supplementary Materials

The following supporting information can be downloaded at: https://www.mdpi.com/article/10.3390/jcm13195805/s1.
