# Supplementary material for: Combined Effects of Cyclic Hypoxic and Mechanical Stimuli on Human Bone Marrow Mesenchymal Stem Cell Differentiation: A New Approach to the Treatment of Bone Loss
Source: J Clin Med. 2024 Sep 28;13(19):5805. doi: 10.3390/jcm13195805 (PMC11476683; doi:10.3390/jcm13195805)
Supplement: Supplementary file 1 [file jcm-13-05805-s001.zip › jcm-3193920-supplementary.pdf]

SUPPLEMENTARY MATERIAL - WESTERN-BLOT IMAGES

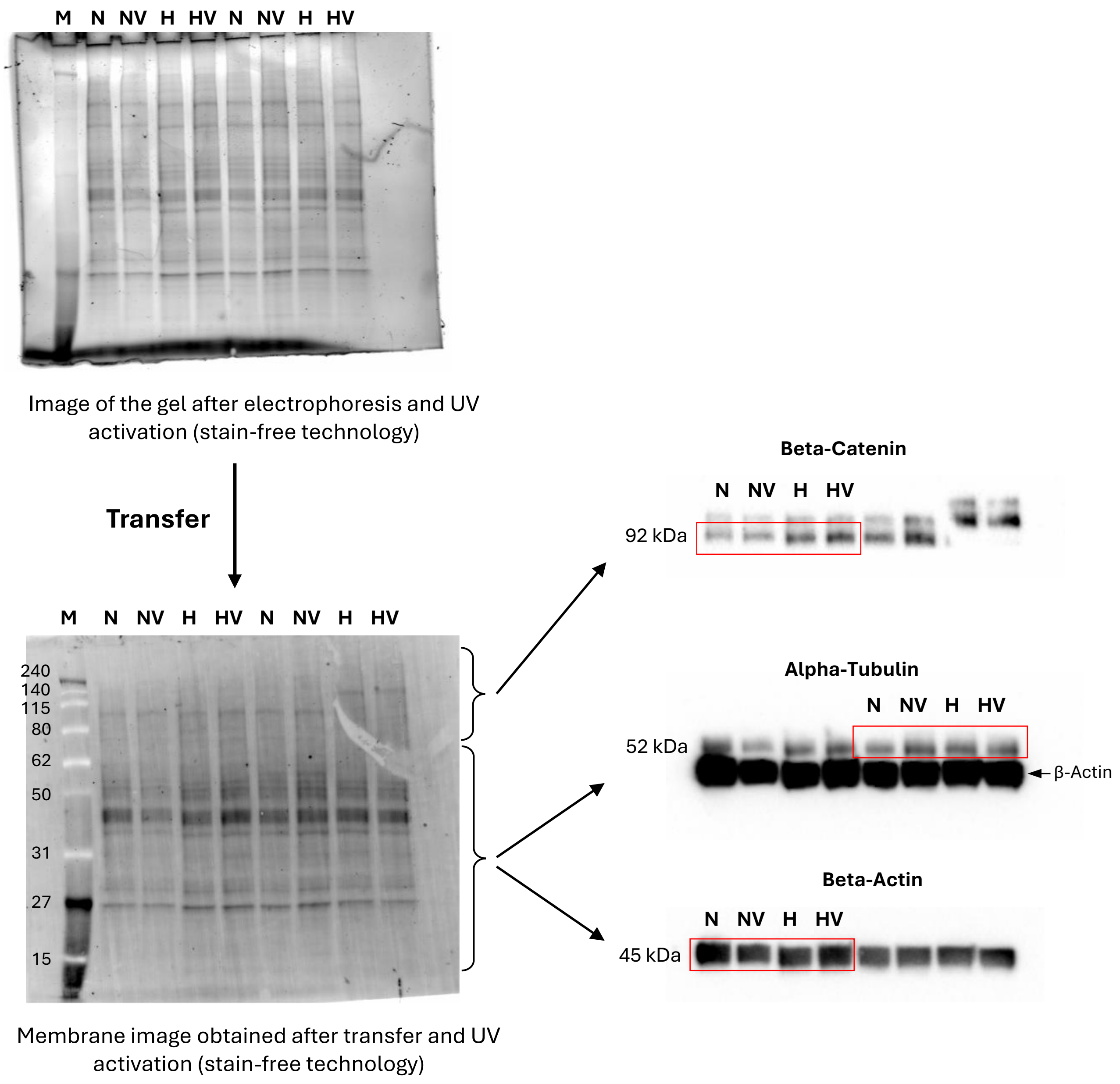

Figure S1. Images corresponding to the wester-blot used for the confection of figure 4 of the manuscript. Extracts from MSC cultures pre-treated under the different experimental conditions (N, NV, H and HV) and subsequently induced to differentiate into osteoblasts for 14 days were separated by electrophoresis in duplicate. The image of the gel was then obtained by UV activation on a ChemiDoc XRS+ Gel Imaging System (Bio-Rad) and of the membrane after transfer. These images were subsequently used to normalise the signal of each of the proteins evaluated with the signal of the total protein loaded per lane, using Image Lab software (Bio-Rad). The PAL-EPL-500 pre-stained ELITE protein Ladder (M) was used as a marker. The bands of which can be observed on the transferred membrane. Once the membrane was transferred, it was divided into two parts by cutting it at approximately the 62 - 80 KDa line. The upper part was incubated with the anti-beta catenin antibody (92 kDa) and the lower part was incubated first with an anti-beta actin antibody (45 kDa) and then with an anti-alpha tubulin antibody (52 kDa) (see details of the antibodies in the Material and Methods section of the manuscript). The bands included in the red box are those used for the preparation of figure 4 of the manuscript.

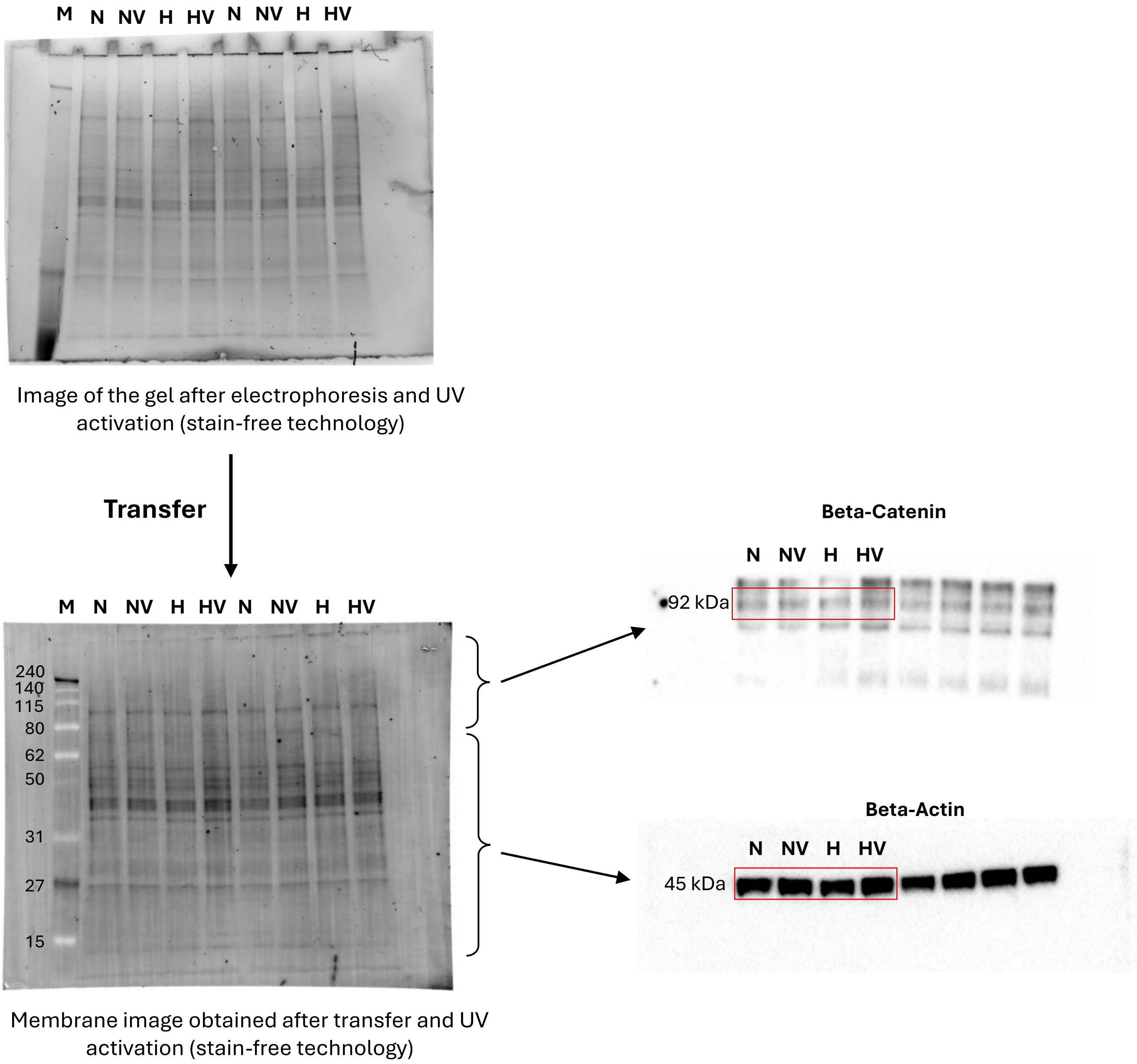

Figure S2. Images corresponding to the wester-blot used for the confection of figure 6 of the manuscript. Extracts from MSC cultures pre-treated under the different experimental conditions (N, NV, H and HV) and subsequently induced to differentiate into adipocytes for 14 days were separated by electrophoresis in duplicate. The image of the gel was then obtained by UV activation on a ChemiDoc XRS+ Gel Imaging System (Bio-Rad) and of the membrane after transfer. These images were subsequently used to normalise the signal of each of the proteins evaluated as described in Figure S1. Once the membrane was transferred, it was divided into two parts by cutting it at approximately the 62 - 80 KDa line. The upper part was incubated with the anti-beta catenin antibody (92 kDa) and the lower part was incubated with an anti-beta actin antibody (45 kDa) (see details of the antibodies in the Material and Methods section of the manuscript). The bands included in the red box are those used for the preparation of figure 6 of the manuscript.

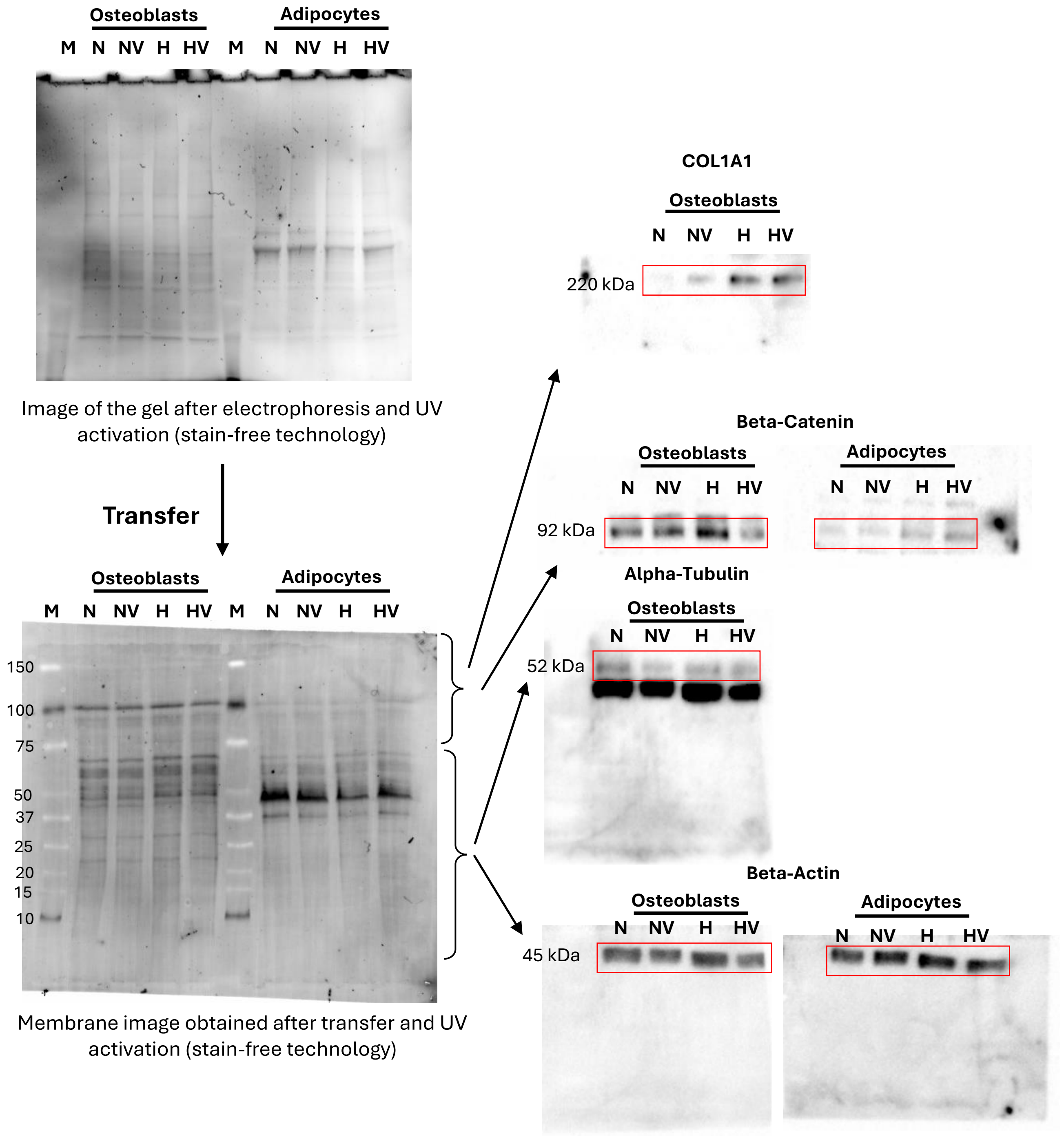

Figure S3. Images corresponding to the wester-blot used for the confection of figure 8 and 10 of the manuscript. Protein extracts from MSC induced to differentiate into osteoblast or adipocytes under the different experimental conditions (N, NV, H and HV) for 14 days were separated by electrophoresis. The image of the gel was then obtained by UV activation on a ChemiDoc XRS+ Gel Imaging System (Bio-Rad) and of the membrane after transfer. These images were subsequently used to normalize the signal of each of the proteins evaluated as described in Figure S1. The protein ladder (M) used in this electrophoresis was Precision Plus Protein Dual Color (Bio-Rad). Once the membrane was transferred, it was divided into two parts by cutting it at approximately the 75 KDa line. The upper part was incubated with the anti-COL1A1 antibody (220) and anti-beta catenin antibody (92 kDa). The lower part was incubated first with an anti-beta actin antibody (45 kDa) and then with an anti-alpha tubulin antibody (52 kDa) (see details of the antibodies in the Material and Methods section of the manuscript). The bands included in the red box are those used for the preparation of figures 8 and 10 of the manuscript.

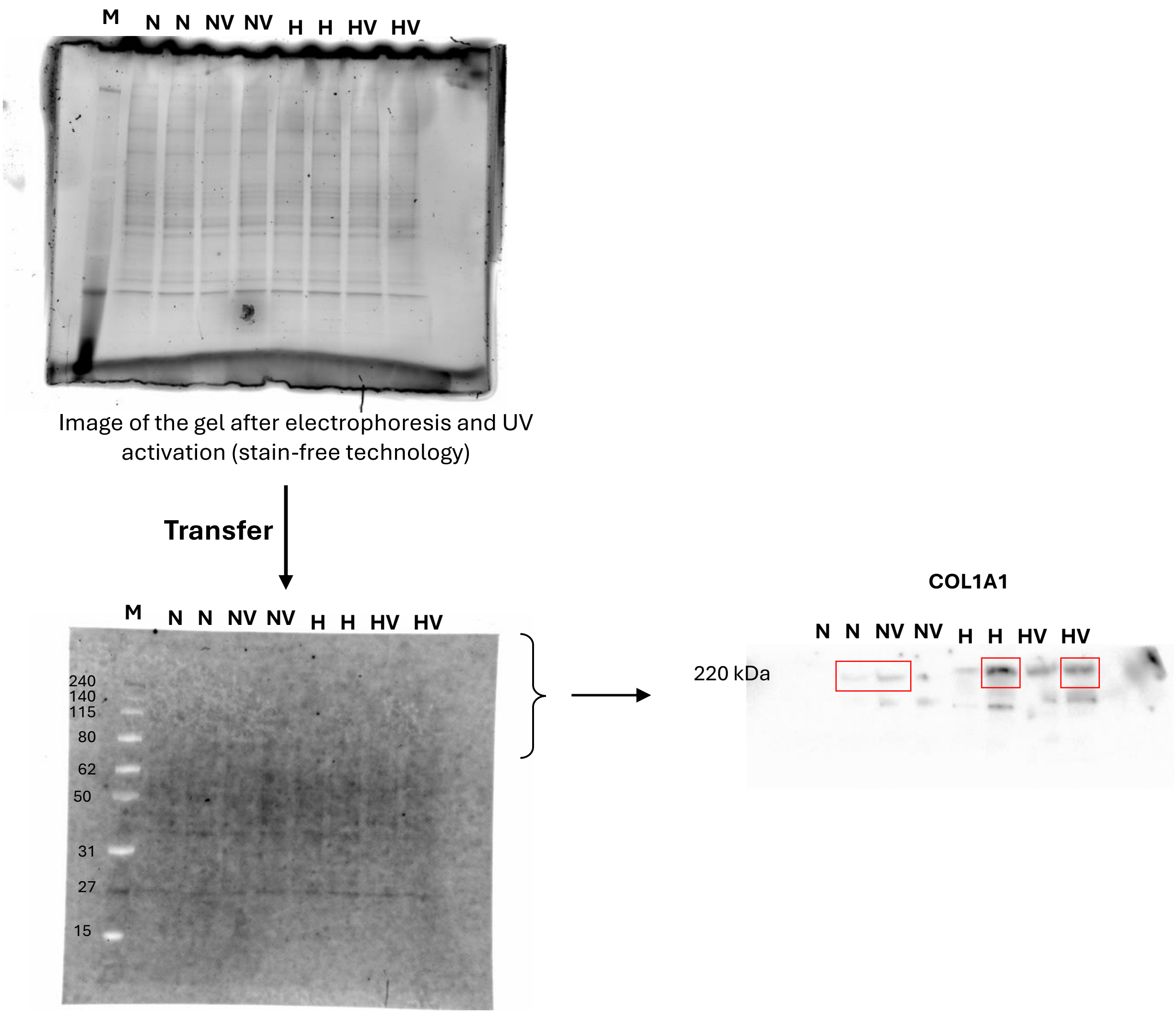

Figure S4. Images corresponding to the wester-blot used for the confection of figure 4b (COL1A1). Extracts from MSC cultures pre-treated under the different experimental conditions (N, NV, H and HV) and subsequently induced to differentiate into osteoblasts for 14 days were separated by electrophoresis in duplicate. The image of the gel was then obtained by UV activation on a ChemiDoc XRS+ Gel Imaging System (Bio-Rad) and of the membrane after transfer. These images were subsequently used to normalize the signal of each of the proteins evaluated as described in Figure S1. The PAL-EPL-500 pre-stained ELITE protein Ladder (M) was used as a marker. The bands of which can be observed on the transferred membrane. Once the membrane was transferred, it was divided into two parts by cutting it at approximately the 62 - 80 KDa line. The upper part was incubated with the anti-COL1A1 (220 kDa).The bands included in the red box are those used for the preparation of figure 4 of the manuscript.
